# Developing and Evaluating JIApp: Acceptability and Usability of a Smartphone App System to Improve Self-Management in Young People With Juvenile Idiopathic Arthritis

**DOI:** 10.2196/mhealth.7229

**Published:** 2017-08-15

**Authors:** Ran A Cai, Dominik Beste, Hema Chaplin, Socrates Varakliotis, Linda Suffield, Francesca Josephs, Debajit Sen, Lucy R Wedderburn, Yiannakis Ioannou, Stephen Hailes, Despina Eleftheriou

**Affiliations:** ^1^ Arthritis Research UK Centre for Adolescent Rheumatology University College London London United Kingdom; ^2^ Department of Computer Science University College London London United Kingdom; ^3^ University College London Hospitals NHS Foundation Trust Adolescent Rheumatology London United Kingdom; ^4^ UCL Great Ormond Street Institute of Child Health Infection, Immunity, Inflammation, and Physiological Medicine London United Kingdom

**Keywords:** juvenile idiopathic arthritis, self-management, adolescent, young adult, mobile applications, qualitative research, smartphone

## Abstract

**Background:**

Flare-ups in juvenile idiopathic arthritis (JIA) are characterized by joint pain and swelling and often accompanied with fatigue, negative emotions, and reduced participation in activities. To minimize the impact of JIA on the physical and psychosocial development and well-being of young people (YP), it is essential to regularly monitor disease activity and side effects, as well as to support self-management such as adherence to treatment plans and engagement in general health-promoting behaviors. Smartphone technology has the potential to engage YP with their health care through convenient self-monitoring and easy access to information. In addition, having a more accurate summary of self-reported fluctuations in symptoms, behaviors, and psychosocial problems can help both YP and health care professionals (HCPs) better understand the patient’s condition, identify barriers to self-management, and assess treatment effectiveness and additional health care needs. No comprehensive smartphone app has yet been developed in collaboration with YP with JIA, their parents, and HCPs involved in their care.

**Objectives:**

The objective of this study was to design, develop, and evaluate the acceptability and usability of JIApp, a self-management smartphone app system for YP with JIA and HCPs.

**Methods:**

We used a qualitative, user-centered design approach involving YP, parents, and HCPs from the rheumatology team. The study was conducted in three phases: (1) phase I focused on developing consensus on the features, content, and design of the app; (2) phase II was used for further refining and evaluating the app prototype; and (3) phase III focused on usability testing of the app. The interview transcripts were analyzed using qualitative content analysis.

**Results:**

A total of 29 YP (aged 10-23, median age 17) with JIA, 7 parents, and 21 HCPs were interviewed. Major themes identified as the ones that helped inform app development in phase I were: (1) remote monitoring of symptoms, well-being, and activities; (2) treatment adherence; and (3) education and support. During phase II, three more themes emerged that informed further refinement of the app prototype. These included (4) adapting a reward system to motivate end users for using the app; (5) design of the app interface; and (6) clinical practice integration. The usability testing during phase III demonstrated high rates of overall satisfaction and further affirmed the content validity of the app.

**Conclusions:**

We present the development and evaluation of a smartphone app to encourage self-management and engagement with health care for YP with JIA. The app was found to have high levels of acceptability and usability among YP and HCPs and has the potential to improve health care and outcomes for this age group. Future feasibility testing in a prospective study will firmly establish the reliability, efficacy, and cost-effectiveness of such an app intervention for patients with arthritis.

## Introduction

Juvenile idiopathic arthritis (JIA) is one of the most common chronic diseases with an early onset in childhood or adolescence. It causes articular inflammation, which in turn leads to pain, swelling, and stiffness of the joints [1]. The progression of JIA into adulthood is common in the majority of teenagers with JIA [2] and, if not treated effectively during the childhood and adolescent years, it can lead to disability and complications related to educational, psychosocial, and physical development in later life [3]. There are now effective therapies for JIA that have the potential to transform the quality of life for young people (aged 10-24 years; YP) [4] with the disease [1]. If optimal treatment is instigated, it can improve JIA prognosis and negate the need for further health care input such as joint replacement surgery, which was often required in YP before the advent of new therapies [5-8].

However, treating JIA requires patients to take regular medications and attend frequent hospital appointments for monitoring disease progression and side effects. In addition, successful treatment often relies on a holistic approach such as engaging YP in appropriate physical exercises and maintaining psychological well-being [9-11]. Health outcomes are thus highly dependent on self-management behaviors to facilitate adherence of YP to treatment protocols and engagement in behaviors that promote physical and emotional well-being [12-15]. As YP mature and transition from pediatric to adult services, they are expected to become more independent and responsible for their own health care. Unfortunately, health service provision often fails to offer the type of support and skills that YP need to manage their JIA, especially during times of significant change [16-18], which may explain the poor adherence to treatment and disengagement with services in YP [19-22]. It is therefore essential for health care professionals (HCPs) to adopt effective and innovative ways to promote self-management promptly and effectively [14,23-26].

According to a model known as the COM-B system [27] that emerged from a systematic review of behavior change theories, behavior (B) may depend on an interaction between three important components: capability (C), opportunity (O), and motivation (M). When one component is not fulfilled, it could impose barriers to self-management; this has been used to explain issues with adherence [28] and applied in several interventions [29,30]. The COM-B model can explain why providing educational information aimed at increasing skills and understanding alone, which was the main focus of previous self-management interventions, has not been sufficient in engaging YP and that additional intervention functions may be necessary [17,31]. These may require sending reminders, monitoring problems and achievements, or offering incentives in ways that are acceptable and appropriate for YP.

An ideal resource-efficient way of improving self-management is by incorporating multiple intervention functions to address all three sources of behaviors through smartphone technology. In general, YP are already familiar and comfortable using smartphone apps in their everyday lives. It is estimated that 75% of adolescents over the age of 13 years in the United Kingdom use smartphones on a daily basis, and smartphone ownership is highest among YP [32]. Capitalizing on the popularity of smartphone-based entertainment in adolescents, health apps may represent an important means of increasing self-management through remote monitoring and access to information [33-36]. For example, health management behaviors can be integrated with daily activities, using technologies that can track information “on the go.” Apps used to monitor symptoms remotely also have considerable potential in helping HCPs deliver safe and timely health care, as it provides more accurate and frequent reports of health status compared with using paper diaries [37-39]. Reliable and secure electronic data collection of symptoms and emotions not only benefits clinical assessments, but it is also an established method for validly collecting daily data in research as well [40-43] and can help advance knowledge on factors influencing disease progression.

However, recent systematic reviews of the literature exploring the effectiveness of mobile apps designed to support the management of chronic physical conditions by YP [44-46] found a limited number of apps in the scientific database. This is in stark contrast to the thousands of commercial apps available that have not been developed in close partnership with end users. Only one app [47-49] has been developed for JIA patients, but it did not involve parents and HCPs in the development process and its function is limited to monitoring symptoms. The app is also targeted for patients in pediatric clinics (<18 years), whose needs and preferences may differ from YP who have already transitioned to adult clinics (18-24 years). Moreover, previous reviews did not identify a single app for YP that designed a clinician interface jointly with the patient app portal, and none had a sound theoretical rationale.

Therefore, this study aimed to address issues with self-management and engagement with health care in YP by developing and evaluating a comprehensive smartphone-based app system for, and in collaboration with, (1) YP with JIA and (2) HCPs involved in their care. We followed a theory- and user-driven approach [50-52], where user feedback helped understand optimal ways of delivering intervention functions that have been identified in the COM-B model to overcome barriers to self-management in YP. This approach is in line with the United Kingdom Medical Research Council (MRC) guidelines, which highlight the importance of identifying appropriate theory to inform the development of a complex intervention [50-52].

## Methods

### Participants

Patients were eligible for recruitment to the study when they fulfilled the following criteria: (1) a diagnosis of JIA, defined as arthritis of unknown etiology lasting more than 6 weeks and of onset at less than 16 years [53]; (2) were aged 10 to 24 years; (3) were able to read and speak English; and (4) were seen at the Great Ormond Street Hospital (GOSH) or University College London Hospital (UCLH) rheumatology clinics. Patients were excluded if they had severe cognitive impairments or major comorbid medical or psychiatric illnesses that would preclude their ability to participate in focus group discussions (FGDs). Parents of patients were eligible if they could read and understand English. Patient demographics and disease-related data collected were age at time of study, age at diagnosis, classification of JIA category, and current medication. The HCPs working in the multidisciplinary team at the two recruiting centers were eligible to participate if they had worked in pediatric/adolescent rheumatology for at least 6 months according to self-report. Participants were able to take part in all three phases of the study. Table 1 summarizes which patient participated multiple times.

### Process

**Figure 1:**
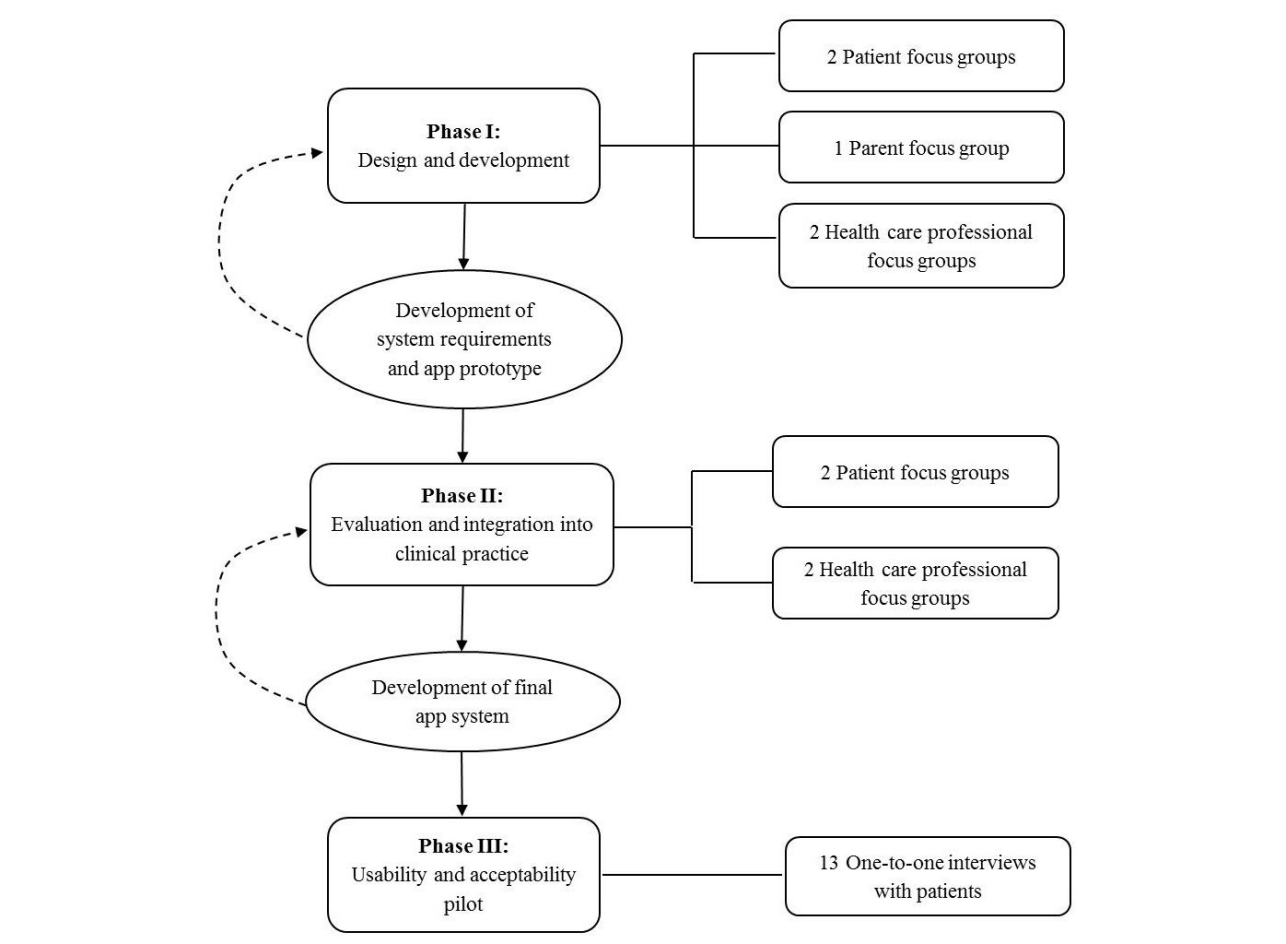
Study phases and iterative development cycle.

The overall project adopted a qualitative, user-centered approach as per the standard published guidance [54-56]. The study was conducted in three phases (see Figure 1). Phase I focused on developing consensus on the features and content of the app. This involved understanding barriers and enablers to self-management in YP, and, as suggested by the MRC guidance [50-52], identifying intervention functions from the COM-B model that should be incorporated in the app. Phase II was used to further refine and evaluate the functionalities and improve the design of the app prototype and the Web application for HCPs. Phase III focused on usability testing of the final app interface for YP. Purposive sampling was used to encompass variations in opinions because of demographic characteristics (age, gender, and ethnicity), disease duration, and JIA subtype. Both YP and parents were approached by a member of the research team in clinics before the rheumatology appointment. The families who agreed to participate gave informed consent or age-appropriate assent with parental consent for those aged under 16 years. Thereafter, YP were offered a group based on their age for phases I and II. The study was approved by the Local Ethics Committee (NRES Committee London – Queen Square, 15/LO/1288).

**Table 1:** Patient baseline characteristics, disease duration, and treatment received.

Phase	Patient ID	Age, in years	Gender	JIA^a^ subclassification	Disease duration, in years	Current medication
I	1	21	F	Persistent oligoarticular JIA	17	Nil. Previously treated with intra-articular steroid injections
	2	18	M	ERA^b^	3	Methotrexate (15 mg/m^2^ sc^c^ weekly)
	3	17	M	Persistent oligoarticular JIA	4	Nil. Previously treated with intra-articular steroid injections
	4	18	M	ERA	7	Etanercept (0.8 mg/kg sc weekly) and methotrexate (15 mg/m^2^ sc weekly)
	5	18	F	Polyarticular JIA	15	Methotrexate (15 mg/m^2^ sc weekly) and adalimumab (40 mg sc fortnightly)
	6	10	M	Extended oligoarticular JIA	4	Methotrexate (15 mg/m^2^ sc weekly)
	7	14	F	Systemic JIA	3	Methotrexate (15 mg/m^2^ sc weekly)
	8	12	M	Extended oligoarticular JIA	7	Nil. Previously treated with intra-articular steroid injections
	9	11	M	Systemic JIA	2	Tocilizumab (8 mg/kg fortnightly) and methotrexate (15 mg/m^2^ sc weekly)
	10	12	F	Psoriatic arthritis	0.4	Methotrexate (15 mg/m^2^ sc weekly)
II	2	18	M	ERA	3	Methotrexate (20 mg/m^2^ sc weekly) and humira (40 mg sc weekly)
	6	10	M	Extended oligoarticular JIA	5	Methotrexate (15 mg/m^2^ sc weekly)
	7	14	F	Systemic JIA	3	Methotrexate (15 mg/m^2^ sc weekly)
	8	12	M	Extended oligoarticular JIA	7	Nil
	9	12	M	Systemic JIA	3	Tocilizumab (8 mg/kg fortnightly) and methotrexate (15 mg/m^2^ sc weekly)
	10	13	F	Psoriatic arthritis	0.8	Methotrexate (15 mg/m^2^ sc weekly)
	11	23	F	Bilateral inflammatory hip arthritis	11	Methotrexate (10 mg/m^2^ sc weekly)
	12	17	F	Systemic JIA	2	Hydroxychloroquine (400 mg orally daily)
	13	20	F	Persistent oligoarticular JIA	5	Methotrexate (20 mg orally weekly)
	14	16	F	Extended oligoarticular JIA	14	Methotrexate (15 mg/m^2^ sc weekly)
	15	17	F	Polyarticular JIA	9	Etanercept (25 mg sc weekly)
	16	14	F	Psoriatic arthritis	12	Adalimumab (40 mg sc fortnightly)
	17	12	F	Persistent oligoarticular JIA	8	Methotrexate (15 mg/m^2^ sc weekly)
III	4	18	M	ERA	8	Methotrexate (15 mg/m^2^ sc weekly)
	18	16	F	Oligoarticular JIA	12	Nil
	19	12	M	Polyarticular JIA	4	Methotrexate (25 mg orally weekly)
	20	14	F	Psoriatic arthritis	8	Methotrexate (12.5 mg/m^2^ sc weekly)
	21	15	M	Extended oligoarticular JIA	8	Naproxen (500 mg twice a day)
	22	17	F	Extended oligoarticular JIA	15	Methotrexate (15 mg/m^2^ sc weekly) and folic acid (5 mg orally weekly)
	23	13	F	Oligoarticular JIA	10	Methotrexate (15 mg/m^2^ sc weekly) and humira (40 mg fortnightly)
	24	15	F	Systemic JIA	3	Methotrexate (27.5 mg/m^2^ sc weekly), anakinra (150 mg sc daily), and folic acid (5 mg orally weekly)
	25	15	F	Oligoarticular JIA	4	Nil
	26	23	M	Extended oligoarticular JIA	21	Keppra (500 mg twice a day)
	27	15	F	ERA	3	Methotrexate (15 mg/m^2^ sc weekly) and folic acid (5 mg orally weekly)
	28	16	M	Polyarticular JIA	1	Methotrexate (15 mg orally weekly) and folic acid (5 mg orally weekly)
	29	18	F	Polyarticular JIA	2	Enbrel (50 mg sc weekly) and methotrexate (7.5 mg orally weekly)

^a^JIA: juvenile idiopathic arthritis.

^b^ERA: enthesitis-related arthritis.

^c^sc: subcutaneously.

#### Phase I: Design and Development

For phase I, we used a nominal group technique to develop consensus on the app content and features. This included a number of separate FGDs for HCPs, younger patients (aged 10-15 years) and their parents, and older patients (aged 16-24 years). Following an introductory presentation on the overall goals of the meeting, YP and parents were asked what features and information will help them or their child better follow their treatment plans and cope with arthritis-related symptoms and consequences. The HCPs were asked what data can be collected remotely to help with understanding and assessing patient’s condition and treatment effectiveness. All participants were also questioned regarding the design and aesthetics of the user interface. Lists of questions posed to each of the groups are summarized in Multimedia Appendix 1. Consensus was considered as having been achieved when 75% of the participants endorsed a given answer. Answers that did not reach 75% endorsement were discarded or reformulated through discussion.

Participants’ answers to the questions posed during phase I FGDs were used to generate a catalog of initial system requirements. An app prototype was then developed in collaboration with the Computer Science Department at UCLH. The agreed set of features were iteratively built into the app codebase by a developer from the Computer Science Department. When clarification of the data generated was required, participants were queried by email and phone to provide further feedback (see Figure 1). The prototype was developed using open frameworks for cross-platform compatibility and runs on both the Google Android (version 4.4.1 and above) and the Apple iOS (version 8 and above) operating systems.

#### Phase II: Evaluation and Integration Into Clinical Practice

The FGDs were again conducted with HCPs and YP with JIA during phase II to vet and refine the requirements of the phase I app prototype. Participants were able to test and navigate through the app’s various features using a smartphone to improve their understanding of the app and focus on their recommendations. Screenshots of important pages in the app were also shown to examine whether YP and HCPs liked or disliked the design and the question format. They were asked to provide further suggestions for improvement, and technical problems with the app were also recorded.

Both HCPs and YP were also asked whether the information collected would be useful during consultations, and how clinically relevant information reported by patients should be imported to, summarized, and displayed in a Web application for HCPs. Also, what data recorded by patients and information on app usage should be stored for research were discussed. Semistructured interview questions that were developed based on the Usefulness, Satisfaction, and Ease of Use questionnaire [57] were used to guide the FGDs (see Multimedia Appendix 1). The design of the app was modified and new screenshots were generated and evaluated until no further changes were suggested. All FGDs were conducted with each participant group until point of data redundancy or point of no proposed new data [58] and lasted between 60 and 90 min. System analysis and final design were done in joint effort with the developer from the Computer Science Department.

#### Phase III: Usability and Acceptability Pilot

Following the development of the final app, the usability and acceptability of the user interface, as well as the registration and data extraction process were tested with YP during phase III. The interviews with YP took place on a one-to-one basis and YP were shown the app on a smartphone. The researcher explained that the purpose of the study was to pilot ease of use and evaluate its features and encouraged YP to input information in all sections of the app at least once. After navigating the various features of the app and using it for 10 to 15 min, YP answered a usability and acceptability questionnaire [57] where they rated on 5-point scales their general impressions of the app, its user-friendliness, clarity of information provided, and whether or not they found the app useful and would recommend it to other YP (see Multimedia Appendix 2). In addition, YP answered qualitative questions regarding the specific features of the app, and how it can help them improve self-management (see Multimedia Appendix 1). Each interview lasted between 25 and 45 min. Discussions were audio-recorded for all phases of the project, and field notes were made for phase III. At all FGDs, RAC and HC were present as interview leader and observer, respectively.

### Data Analysis

All audio recordings were transcribed and qualitative content analysis [59,60] was applied. Qualitative data from transcripts and field notes were carefully reviewed to develop a coding system that reflected perceptions and suggestions for the smartphone-based JIA app. Data were then assigned with codes based on their content. Codes with similar content were grouped into meaningful categories. RAC and HC read and analyzed the transcripts separately and then compared the results. Differences from the separate analyses were discussed until consensus was reached. The coding system and analytic process were further discussed with DE, an experienced pediatric rheumatologist. The final analysis organized the categories together into overarching themes that were developed and refined by discussions between RAC, HC, and DE. Discussions were also held between the researchers on how each category mapped onto the COM-B model. In addition, content analysis was performed according to the age group of YP (10-15 years and 16-24 years) to identify any age-specific differences in opinion. All qualitative data were analyzed using NVivo software (NVivo version 10, QSR International, 2012) [61].

## Results

### Participant Characteristics

For phase I, a total of 10 patients were recruited and were separated into two groups: group A included 5 patients (median age 12 years, range 10-14 years, 2 females) and group B included 5 patients (median age 18 years, range 17-21 years, 2 females). A separate FGD was conducted with 7 parents of patients from group A. Two patient FGDs were again conducted for phase II: group A included 7 patients (median age 12 years, range 10-14 years; 4 females) and group B included 6 patients (median age 18 years, range 16-23 years, 5 females). Phase III interviewed 13 YP. Demographics and disease characteristics of YP included in the study are shown in Table 1. The participants had a variety of JIA categories with a median time since diagnosis of 4 years (range 5 months-17 years) for patients in phase I, 5 years (range 10 months-14 years) for patients in phase II, and 8 years (range 1-21 years) for patients in phase III. The participants were also diverse in ethnicity (62% white, 24% Asian, 10% black, 3% Hispanic).

Two FGDs with HCPs were also conducted for phases I and II. For phase I, a total of 19 HCPs with a median of 7 years (range 1-20 years) of experience in pediatric/adolescent rheumatology participated. These HCPs included 7 medical consultants, 4 clinical nurse specialists, 4 rheumatology trainees, 2 research nurses, 1 physiotherapist, and 1 clinical psychologist. For phase II, a total of 15 HCPs with a median of 8 years (range 1 to 20 years) of experience participated. The group included 6 medical consultants, 3 rheumatology trainees, 2 clinical nurse specialists, 2 research nurses, and 2 research fellows.

### Phase I: Design and Development

**Figure 2:**
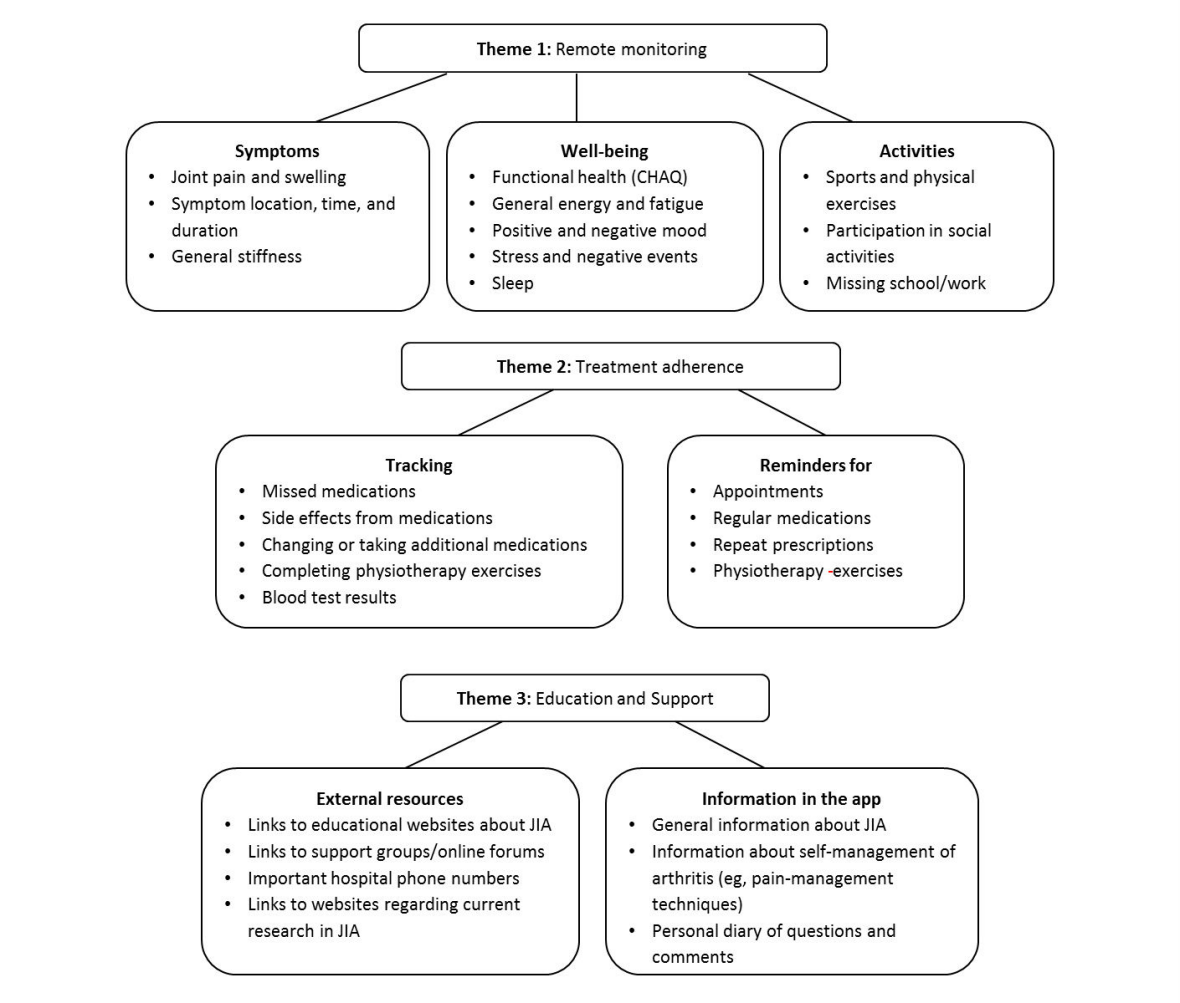
Results of content analysis of transcripts from focus groups for phase I.

It was apparent that YP with JIA wanted a multifaceted way to help them manage their condition. Transcript analysis of data collected during phase I revealed three distinct themes that guided the development of this app (see Figure 2).

#### Theme 1: Self-Monitoring

The first theme was self-monitoring, as all participants thought that tracking certain information will (1) help them understand what influences their JIA symptoms and (2) provide better data for HCPs to use during assessments. This theme included six initial categories for monitoring; however, only those for which there was >75% agreement on its importance among YP, parents, and HCPs were included in the app. These were monitoring (1) symptoms, (2) general well-being, (3) activities, and (4) sleep. Categories that did not achieve this concordance were related to tracking (5) weather and (6) nutrition.

All participants identified tracking of joint symptomatology, including stiffness, swelling, and pain as very important, particularly as this varies in intensity, location, and duration over time. They felt that using the app would reduce recall bias and enable both patients and clinicians to monitor symptoms in real time:

I want to note the dates of when I have symptoms before I forget them, so that I can show the doctors, and help them better understand my illness and what is really going on.

Patient 3

One patient stated:

Daily monitoring of pain is better than giving a monthly average at the clinic.

Patient 2

Participants indicated that the app should include continuous visual analog scales (VAS) to indicate the intensity of JIA-related symptoms by moving a bar across a horizontal line in preference to categorical selections. One adolescent stated:

Instead of choosing just one number, I want to be able to choose a range between the numbers, like between 6 to 7.

Patient 3

As requested by YP, each scale was accompanied with a cartoon face that changed facial expressions from extremely positive at the left to extremely negative at the right. Psychometric properties of the VAS have been widely explored with children as young as 8 years of age and have confirmed its test-retest reliability, construct validity, and criterion-related validity [62,63].

**Figure 3:**
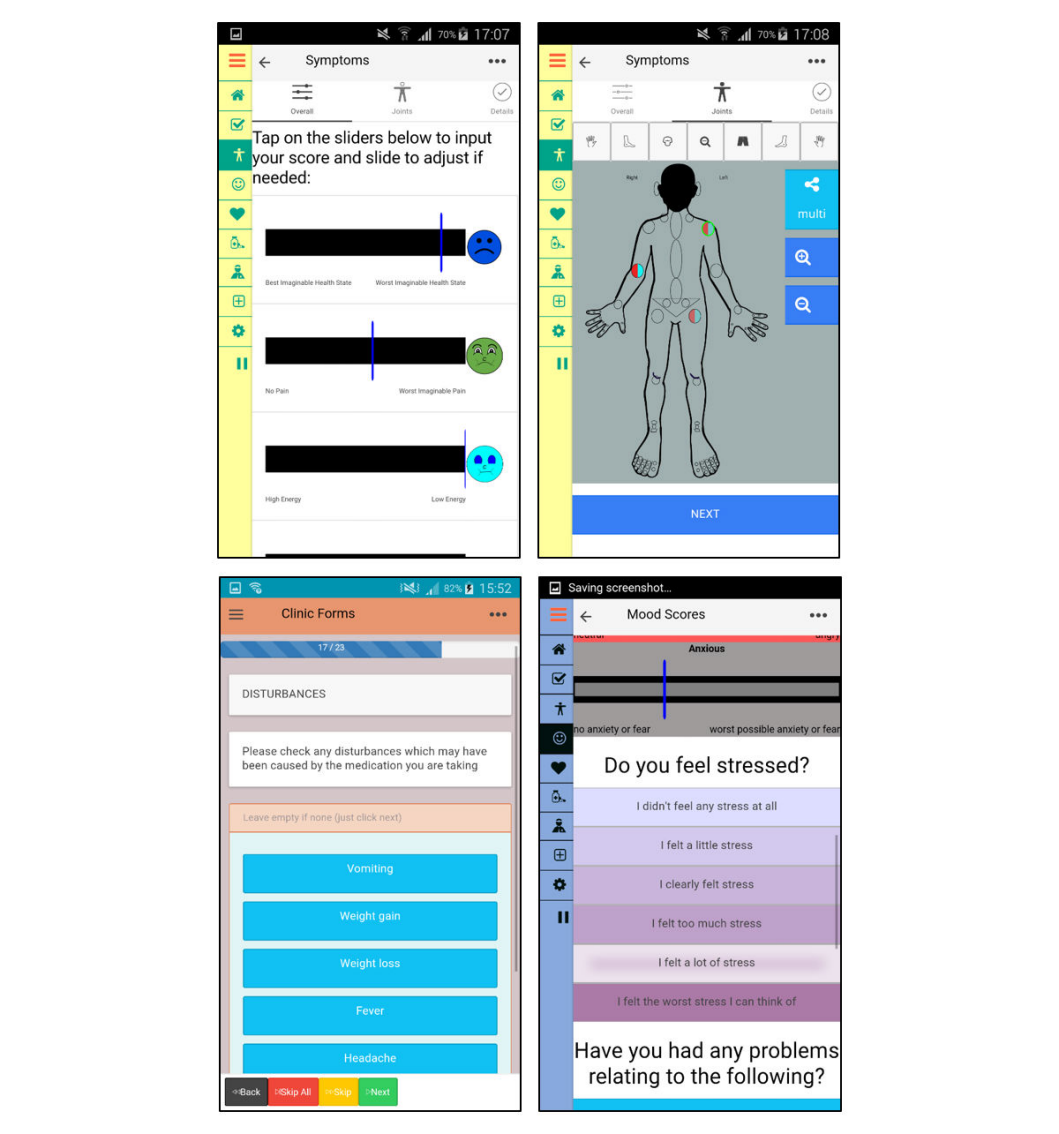
Screenshots of the app for monitoring symptoms, thoughts, & feelings.

Additionally, YP wanted to use a body map to indicate how painful and swollen a specific joint is. Body maps can facilitate children’s reports of location of pain [64,65], and electronic versions have been used successfully with children as young as 8 years old [40]. The app will include a simple body map (see Figure 3) with circles at major joint areas in the body, mirroring what is used in routine clinical assessments [66]. YP can click on a joint that is painful or stiff and use a VAS to indicate the intensity of their pain and stiffness, and the joint’s color will then change accordingly. This is important to include, as YP often experience different pain intensities from different joints, and their stiffness intensity also vary by location and time of day. Both YP and HCPs thought that recording this will help them better explain their symptoms and assess whether treatment is required (eg, physiotherapy exercises targeting specific joints). Lastly, YP requested an open text area where they can input additional details regarding their symptoms and add comments about the type of pain, with one of the patients stating the following reason:

It’s about how you feel and how you describe it instead of just having a number.

Patient 4

Being able to monitor general well-being such as mood, fatigue, and functional health were also identified as important. For example, YP indicated:

Tracking stressful events and mood and how that impacts your symptoms is most important.

Patient 4

Three positive (excited, energetic, happy) and three negative emotions (anxiety/fear, sad, angry) were chosen together with YP and HCPs, which were adapted from the Positive and Negative Affect Schedule for Children [67]. These were assessed using the VAS and the horizontal line ranged from “not at all” on the left to “extremely” on the right. A stress scale was also included, but a verbal rating scale for stress (VRSS) was preferred by YP instead of a VAS. The VRSS was found to be more reliable than the VAS and can be easily understood and completed by children as young as 7 years old [68]. It measures stress on a scale from zero to five with responses describing an increase in stress levels. In addition, YP wanted an electronic version of a JIA-related childhood health-assessment questionnaire (CHAQ) [69], which is a widely used functional health status measure in YP with JIA and is administered during routine clinic consultations. They recognized that completing it once every 3 to 6 months during clinic consultations may provide unreliable responses, as it does not reflect variations over time:

I’d like to track more precisely because the answers to those questionnaires can change every day, or even every hour.

Patient 10

Digital CHAQs have been developed and tested in previous studies [63] and were found to yield similar outcomes when compared with the paper form of CHAQ.

Recording activities such as participation in school and physical exercise and sports was another important area for YP. One YP said,

Exercise usually makes me feel better afterwards and doing sports takes the pain away.

Patient 8

Another stated,

I want to be able to write down what type of exercise I did, such as swimming.

Patient 1

YP suggested that the app should provide visual feedback of their data so that they can see how different factors impacted their condition, and what techniques were helpful in managing their symptoms.

#### Theme 2: Treatment Adherence

Participants were interested in using the app to improve treatment adherence by setting reminders for when to take regular medications, request repeat prescriptions, complete physiotherapy exercises, and attend hospital appointments for important assessments and tests (eg, regular blood tests). One of the patients said,

Sometimes when life gets busy I do forget, so having a reminder would be really useful.

Patient 4

**Figure 4:**
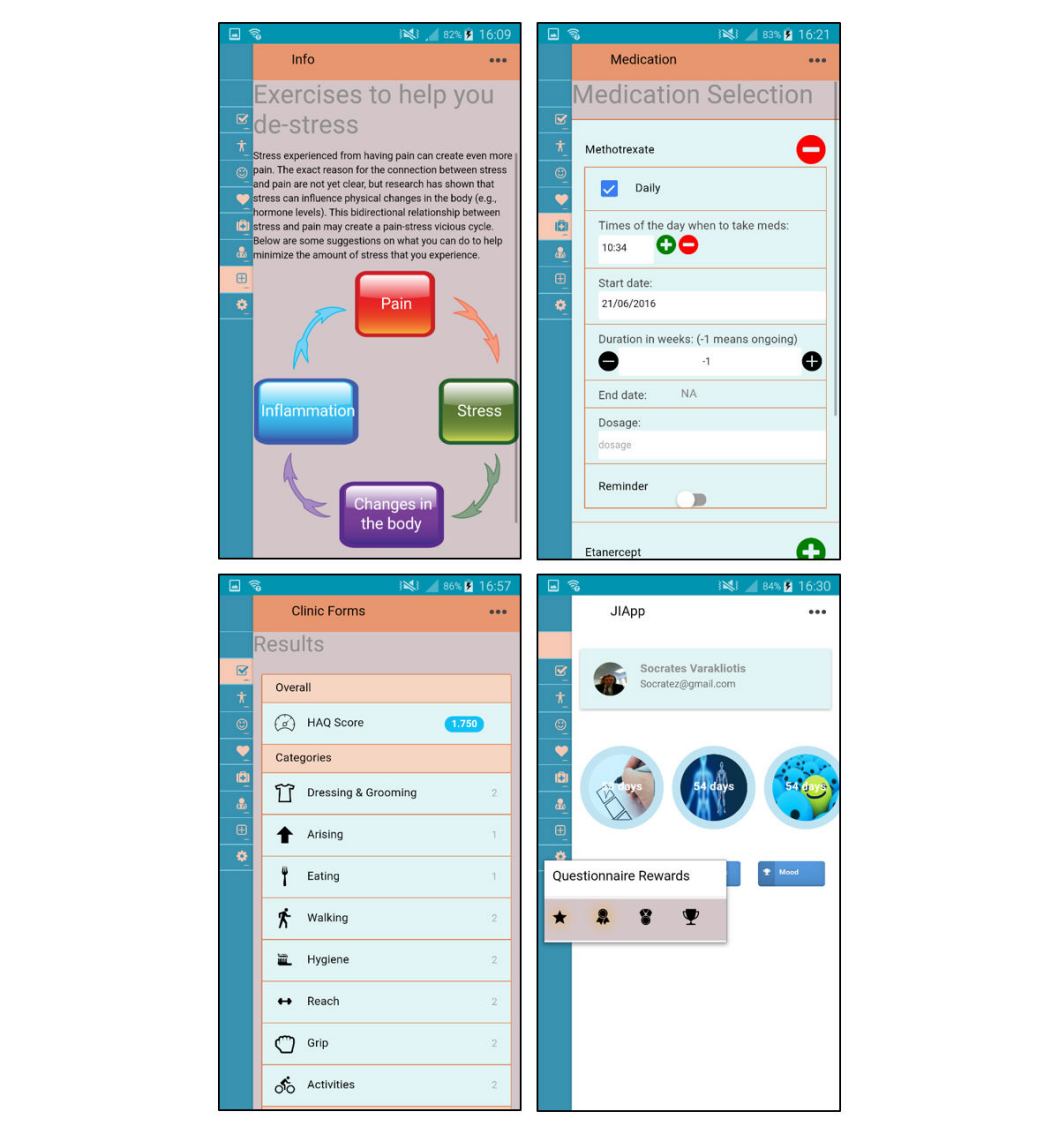
Screenshots of the app for adherence and information.

However, some participants felt that these reminders should be optional to avoid any disengagement with the app because of annoyance from compulsory reminders. Furthermore, YP were interested in recording when medication doses were missed, reasons for missing doses, side effects, medication change, and whether they took any additional medicines such as analgesics or antibiotics and anti-emetics (see Figure 4). Regarding physiotherapy exercises, YP could select which ones they were assigned or would like to perform from a list of possible exercises recommended by physiotherapists at GOSH and could input the number of sets and repetitions. Additionally, both patients and HCPs wanted a section where patients could input their blood test results, with an indication of whether these results were normal and what the tests measured:

I think it’s interesting to have your blood results on the app, together with the doctor’s comments such as there’s nothing to worry about or that something might be a problem.

Patient 2

#### Theme 3: Education and Support

Besides wanting to learn more about their condition, YP wanted to have a discussion forum in the app with other patients. Also, YP from all age groups highlighted the need to have easy access via the app to condition- and treatment-related information, news about research, and social support:

The app should have information on arthritis…also news about research, involvement, and being able to talk to other people.

Patient 2

Additionally, YP said that learning different ways of managing their stress and pain from peers, and knowing that they are not alone, will make them feel more empowered and confident. Moreover, YP mentioned that their arthritis symptoms tend to worsen during times of stress and exams and would like information on how to better manage stressful situations and pain. Therefore, the app will include general information regarding JIA, medications, physiotherapy exercises, and practical coping advice, with links to more detailed and trusted Web resources (see Figure 4). It will also signpost users to information regarding support groups that are reputable and age-appropriate, as well as possible involvement activities organized by local organizations and charities.

Older patients (18-24 years) requested additional information on how to become more independent as they become an adult and what support is available for higher education and work. The app will provide general information covering these topics, with links to how other YP with JIA overcame challenges of growing up. In addition, there is a diary section where YP can write down any questions or comments that they have and can easily refer to during consultations.

### Phase II: Evaluation and Integration Into Clinical Service

**Figure 5:**
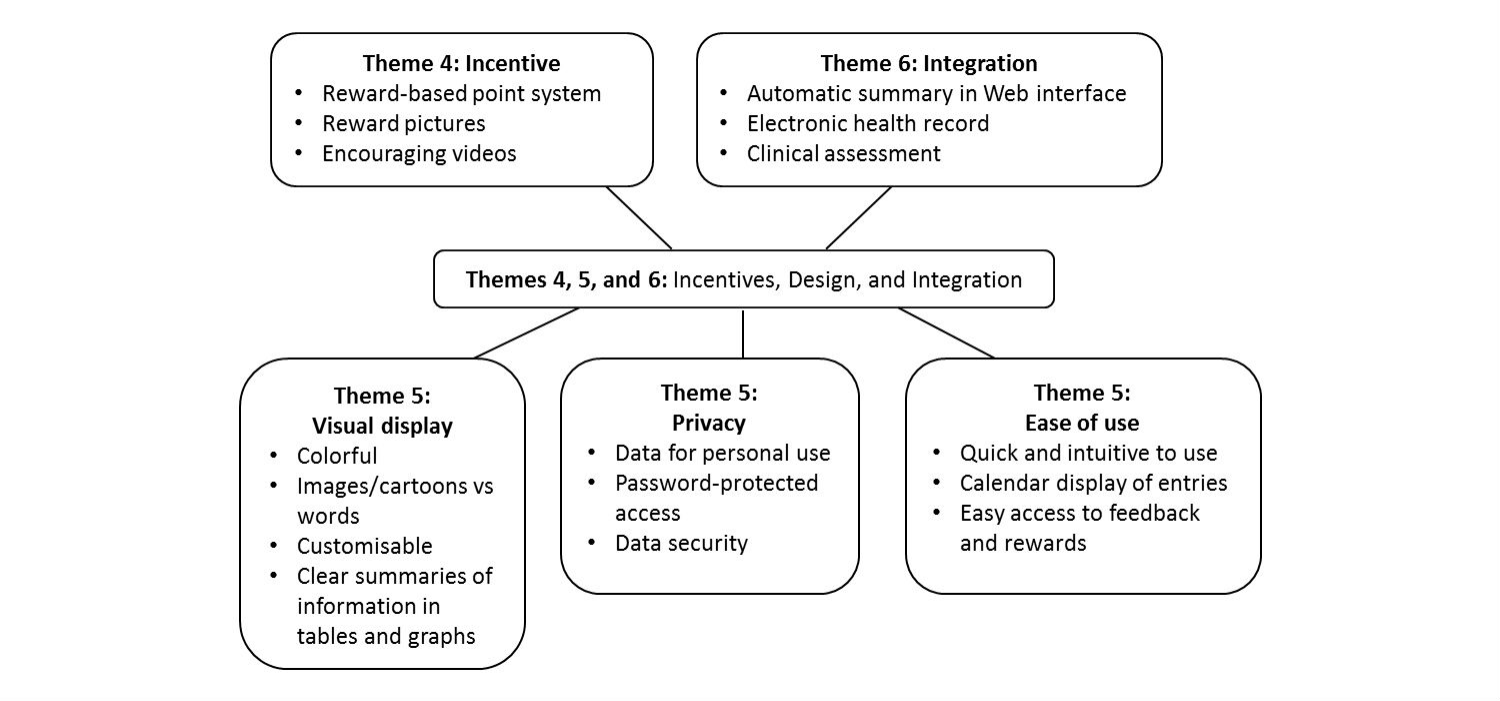
Results of content analysis of transcripts from focus groups for phase II.

During phase II, three more themes emerged (see Figure 5), which related to incentives, design, and integration.

#### Theme 4: Incentives

It was observed that YP wanted to be rewarded for using the app, and they preferred a reward-based rather than a punitive system. One of the patients stated:

You should get points to buy rewards for using the app but you shouldn’t get points taken away, even if you don’t use it for a while.

Patient 6

Therefore, the app will incorporate a point-based reward system and include encouraging video messages from celebrities and athletes. However, this was more appealing to the younger group rather than the older group. Older group enjoyed choosing reward pictures (eg, of landscapes, animals, and sports) to use as their wallpaper and motivational quotes that they could display on the home page.

#### Theme 5: Design of Patient Interface

In terms of designing the user interface, three aspects were important for YP and HCPs: (1) data security, (2) visual appeal/clarity, and (3) convenience. First, as the app will contain patient’s personal details, it needs to be password-protected:

Arthritis is quite personal so some things you might not want to share with other people.

Patient 6

Also, YP wanted the option of diarizing information that would not necessarily be shared with clinicians:

Some things might be a bit personal, and just for me to look at in a diary format.

Patient 13

Second, YP wanted a colorful visual display, easy-to-read fonts, and pictures and were keen on being able to personalize colors and backgrounds. One participant stated,

It’s important to have pictures and as little words as possible.

Patient 3

Participants also suggested that scales should include faces to represent different pain or fatigue levels:

Using faces and cartoon will brighten it up a bit

Patient 4

Moreover, YP wanted more space on the home page to display reward videos and pictures on the bottom; therefore, the home page displays a dial (instead of a drop-down ladder) to count down the number of days that are left until the next weekly deadline (see Figure 4).

Lastly, there was a consensus among YP that it should typically take less than 10 min to input all required information:

Input must be very quick to do and not feel like a chore.

Patient 12

Moreover, YP felt that there needed to be an agreed minimum input to provide clinicians with interpretable data. One adolescent stated:

Even if you have no pain, you can just indicate “no pain” so that doctors know that you didn’t just forget about the app.

Patient 10

It was agreed by both YP and HCPs that the most important data to collect were arthritis-related symptoms, CHAQ, and mood, which should be provided at least once a week. The frequency of completion of other sections should be decided by the YP themselves to give them control over the process.

Ease of use was thus improved in several important ways. First, YP were now able to access the three main monitoring components of the app (symptoms, CHAQ, and mood) directly from the home page rather than from the menu bar. Second, after submitting each input, YP were presented with a pop-up showing how many days ago was their last input for each of the other section, which allowed them to quickly select another section that had not been completed recently. Similarly, hyperlinks were added within the app itself to facilitate access to relevant tips and advice. YP confirmed that these changes made inputting data and finding relevant information faster and more convenient. Lastly, YP requested a calendar display of entries, and the ability to input data retrospectively. One patient said,

Sometimes we have a bad day and we’re tired so we don’t want to the use the app, but we might want to go back and re-enter it later.

Patient 1

All the themes and their associated app functionalities were mapped onto the COM-B model (see Table 2).

#### Theme 6: Clinical Practice Integration

**Figure 6:**
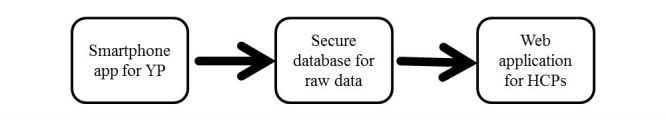
JIApp system.

The HCPs discussed how JIApp can be integrated seamlessly into clinical practice and how reports from patients will be received. Clinically relevant data collected from the app will be automatically aggregated and presented in table and graphical summaries (see Figure 4) on a clinician Web interface. The interface was optimized for usability and data are presented in an intuitive format such that any changes over time or associations between various factors (eg, pain and mood) are visually presented to aid comprehension. Figure 6 illustrates the system, where raw data recorded by YP are stored and clinically relevant information can be summarized and displayed on the clinician interface.

The within-application workflow was also aligned with clinician’s regular work streams. For example, HCPs can access the Web interface via a patient’s existing electronic health records, which can increase the likelihood of integration and access from all multidisciplinary members of the rheumatology team. Information can also be downloaded by HCPs and used to populate clinic letters.

The HCPs also identified possible barriers to integration such as accessibility to all patients and demands for additional resources. For example, HCPs proposed that a desktop version of the app will be beneficial to allow access to YP without a mobile phone. In addition, HCPs were concerned about the additional time needed to monitor remote symptom deteriorations and interpret additional information collected from the app. It was agreed that HCPs are only expected to review the data during or right before a clinic consultation. Therefore, before downloading the app, participants will be informed that data will not be monitored in real time for this stage of the project but will be reviewed during clinic consultations. This is also explicitly explained on the app with a disclosure statement. The HCPs suggested that the effect of app use on HCP-patient interactions and cost-effectiveness of the intervention should be formally assessed in the next stages of the project during a prospective pilot study.

**Table 2:** Applying the Capability, Opportunity, Motivation-Behavior (COM-B) model to major themes identified for JIApp.

Themes	Capability	Opportunity	Motivation
Theme 1: Self-monitoring	Self-awareness and understanding patterns		Perception of illness and self-efficacy
			Better patient-clinician communication
Theme 2: Treatment adherence		Reminders for treatment adherence	Recording past adherence can stimulate action
Theme 3: Education and support	Knowledge of juvenile idiopathic arthritis and treatments		Confidence in skills and empowerment
	Knowledge of self-management skills and emotion regulation		
Theme 4: Incentives			Expectation of rewards
Theme 5: Design of user interface		Convenient monitoring	
		Convenient access to reliable information and support	

### Phase III: Usability and Acceptability Pilot

All YP endorsed the app and thought that it would be beneficial for them right from the point of initial diagnosis (mean acceptability rating=4.29; standard deviation, SD=0.70). Also, YP were able to input important data within 10 min and found it intuitive to use and easy to navigate (mean usability rating=4.25, SD=0.79).

The usefulness of each of the app functionality was reviewed using qualitative interviews. It was apparent that there were multiple reasons for using the app such as its potential to provide YP with *opportunities* for better self-management, as all YP discussed the importance of having medication reminders and easy access to information, advice, and support. Furthermore, YP commented on how the app made them feel as if all the information they need is now at their “fingertips,” which makes managing their arthritis seem less difficult and increases the likelihood of actually reading the information and engaging in recommended behaviors and self-monitoring. They also appreciated how the information displayed was relevant to their condition and concise. One patient said,

I like the way the sections are organized, and how it is not too overwhelming, but how I do have the option of finding out more if I want to.

Patient 22

In terms of improving *motivation* to self-manage, YP believed that through self-monitoring, they were able to understand the importance of their own behaviors and how factors under their control can influence disease activity:

I never know when I will flare up…so if this can help me see if what I do can “actually” affect my arthritis, then that would be very helpful.

Patient 29

Moreover, YP emphasized how reading about other people’s successes and having a record of their own past success with managing pain and adhering to treatments can increase their confidence in being able to effectively cope with future challenges.

Also, YP were especially interested in doctors acknowledging their input and using data collected from the app to make better assessments and improve their care. They were enthusiastic about integrating the app into their care, as it will make it easier to discuss issues and symptoms that were recorded beforehand:

This app can help me remember what I wanted to say to my doctors, which I often forget by the time I come to the hospital…it will make it easier for me to show them what I mean.

Patient 18

Improving communication and patient-clinician relationship can in turn motivate further engagement with health care. Lastly, YP thought the reward system makes it enjoyable to use and is simple, yet engaging:

The app itself is already beneficial to the users, but the images as rewards is actually pretty nice and simple. I like it.

Patient 4

Most of the YP also fed back on how using the app can improve areas related to self-management *capability*, as reading reliable recommendations regarding pain management techniques will teach them better coping strategies:

Sometimes there’s nothing you can do except deal with it yourself, and this app can help you do that.

Patient 21

Patients also highlighted the fact that recording symptoms the moment they occur using the app enables better understanding of their condition and treatment beyond what can be provided by education alone. One of the patients said,

The app is a good way to keep track of my symptoms and should be given by doctors, especially when you’re first diagnosed.

Patient 4

They believed that each individual is different, and inputting data on a regular basis can help them become aware of their personal triggers and feel more in control of their arthritis:

I like visual information and to see how my stress levels interact with my arthritis.

Patient 26

## Discussion

### Principal Findings

This study presents the design and development of a smartphone app to remotely record symptoms and encourage self-management and engagement with health care for YP of all ages (10-24 years) with JIA. We worked closely with YP, parents, and HCPs to identify a catalog of initial requirements for the app system and the necessary changes needed to improve the functionalities of the app and its ease of use. Phase III usability testing demonstrated high rates of overall satisfaction with the app among YP and further affirmed the theoretical validity of the choices made in its design.

Through this app, YP are now able to record numerous additional parameters relating to their physical and psychological well-being, access relevant educational information and social support forums, receive treatment-related reminders, and improve communication with HCPs. More importantly, clinically relevant data is collected by YP themselves in the real-world environment rather than by the HCPs in the hospital environment and is owned by the patient, thus fostering the concept of independent management of health care as the YP mature [70-72]. Moreover, having a clear theoretical basis allows future studies to identify which functions of the app system are driving improvements in self-management.

From the HCP perspective, this app also has the potential to standardize the way reports of clinical symptoms and medication adherence are captured in natural settings and enable fine-grained monitoring of arthritis in a way that is impossible to obtain with occasional clinic visits. Having a more accurate and holistic overview of the symptoms and problems of YP may improve the dynamics of consultation, as time spent on gathering information can be directed to discussing issues that are of concern to the patient. Such a system may also allow systematic collection of a clinically relevant standardized dataset, intended to ensure uniform data collection in the clinical setting [73] that can then inform NHS commissioning processes such as monitoring the efficacy of biologic therapy and the impact of such therapies on patient’s functional status. We also wish to prospectively assess in the next stages of the project the feasibility of collecting and processing additional mood- and pain-related data for HCPs, and the impact this has on future resource use (efficient use of consultation time, early identifications of problems, and efficient referrals), treatment effectiveness (whether it facilitates joint decision making with YP and the patient-doctor relationship), and, ultimately, health-related outcomes.

From the health care information technology perspective, the effort was not limited to a standalone app. JIApp is the front-end data-collection and patient-interaction component of a larger platform, involving a substantial back-end architecture, which includes (1) a flexible database mechanism that synchronizes cached phone data to the data repository, (2) a YP reward service, (3) user feedback and issue tracking system, and (4) secure transport of data from devices to the back-end. Most importantly, the platform also comprises a Web-based clinician’s interface to facilitate retrieval and presentation of patient data in a suitable and efficient manner for HCPs.

Moreover, this is not purely a bespoke app for GOSH and UCLH Rheumatology, and the development efforts did not limit the system design to this domain. For example, the software component for the questionnaires could generate any form of questionnaire with a variety of answer options such as optional/blank, single, multiple, free-text, slider-based, and so on. One could reuse such components for rapid development and deployment of similar data-oriented health care apps. The front-end JIApp will be made available via popular app stores (Google Play and Apple).

### Comparison With Prior Work

To our knowledge, this study is the first to report a theory-driven and user-centered approach to develop a smartphone-based multifunctional app system for HCPs as well as YP with JIA. Previous JIA-related apps have only focused on monitoring symptoms [47-49] and have not considered how the app can be integrated into clinical practice. It is also the only app developed that addresses all components of the COM-B model for self-management.

Similar self-management apps have been developed for chronic pediatric conditions other than JIA, particularly for YP with diabetes [74,75], asthma [76,77], and cancer [78,79]. Themes that emerged from previous studies were related to what YP with JIA discussed in terms of ease of use, information sharing, rewards, and design. However, medication reminders and tracking missed medications and blood test results were not discussed by YP with other chronic conditions. This shows how certain requirements are unique to JIA patients and highlights the importance of developing condition-specific apps.

In terms of app effectiveness, some asthma and diabetes apps have been shown to benefit YP by increasing asthma control [76] and frequency of blood glucose monitoring [74], whereas others have found no benefits of mobile apps [75,77]. Research in this area is still scarce, and more studies with larger sample sizes and longer follow-ups are needed to evaluate the impact of apps on self-management in YP. However, it is likely for apps that go through iterative development cycles involving both YP and HCPs to be more effective.

### Limitations

Our user groups were diverse, even though relatively small, with participating patients having different JIA subtypes at various stages of their treatment and from different ethnical backgrounds. The app’s design and evaluation were not affected by the characteristics of YP, but participation bias may have influenced the themes that emerged from our FGDs and interviews. For example, all study participants were computer literate and had easy access to smartphones; whether further refinements of the app are necessary for patients who are less familiar with this technology needs to be established. We considered this limitation by using open frameworks to develop the app so that it can be deployed as a desktop version (running on most modern browsers such as Chrome, Safari, and Firefox) with minimal effort. It is important for future studies to evaluate patient’s smartphone usage/ownership and its association with recruitment, app usage, and app satisfaction to ensure that the intervention is suitable and easily accessible for all YP.

### Conclusions

In summary, a qualitative user-centered approach was used to develop the first comprehensive smartphone app for YP with JIA. Next steps will involve long-term feasibility testing of the app to establish its effect on patient’s self-management skills, disease outcomes, and general well-being, as well as the cost-effectiveness of such an intervention. In addition, patient’s app usage will be monitored to assess long-term sustainability and understand reasons for disengagement.

## Appendix

Multimedia Appendix 1 Interview questions.

Multimedia Appendix 2 Usability questionnaire.

## Abbreviations

CHAQ: Childhood Health Assessment Questionnaire
ERA: enthesitis-related arthritis
FGD: focus group discussion
GOSH: Great Ormond Street Hospital
HCP: health care professional
JIA: juvenile idiopathic arthritis
sc: subcutaneously
SD: standard deviation
UCLH: University College London Hospital
VAS: visual analog scales
VRSS: verbal rating scale for stress
YP: young people
