# Supplementary material for: Developing and Evaluating JIApp: Acceptability and Usability of a Smartphone App System to Improve Self-Management in Young People With Juvenile Idiopathic Arthritis
Source: JMIR Mhealth Uhealth. 2017 Aug 15;5(8):e121. doi: 10.2196/mhealth.7229 (PMC5575419; doi:10.2196/mhealth.7229)
Supplement: Multimedia Appendix 1 [file mhealth_v5i8e121_app1.pdf]

Multimedia Appendix 1: Interview questions

Supplementary Table S1 Interview guide for phase I (interviews with young people, children, and parents)

A. How do you think the smartphone application can be used to help you improve the management of your arthritis?

B. What can be put into the application to help you better understand your arthritis?

C. What types of data would you like to input remotely (arthritis related functional scores, pain and general well-being questionnaires, validated measure of arthritis disease activity amongst others)?

D. What should be monitored regularly and discussed with your doctors in order to make clinic visits more efficient (e.g., exercise frequency and intensity; adherence, confidence in self-management)

E. Would you like any reminders (e.g., date of clinic appointments, medication)?

F. In your opinion, what kind of health-related information do you think is important and should be tracked or monitored using a smartphone application?

G. How would you like to track this information?

H. What feedback or reward system would you like to have for using the application?

I. How would you design the app and how would you like it to look like?

J. What would you like to name this application and how would you like its logo to look like?

Supplementary Table S2 Interview guide for phase I (interviews with healthcare professionals).

A. How do you think the smartphone application can be used to help young people with managing their arthritis?

B. What information tracked on the smartphone application will help you better understand their arthritis?

C. What types of data would you like your patients to input remotely (arthritis related functional scores, pain and general well-being questionnaires, validated measure of arthritis disease activity amongst others)?

D. What should be monitored regularly and discussed with your patients in order to make clinic visits more efficient (e.g., exercise frequency and intensity; adherence, confidence in self-management)

E. Do you think patients should get any reminders (e.g., date of clinic appointments, medication)?

F. In your opinion, what kind of health-related information do you think is important and should be tracked or monitored using a smartphone application?

G. How would you like your patients to track this information?

H. How would you like the information entered by patients to be displayed and summarized for your review?

Supplementary Table S3 Interview guide for phase II (interviews with young people)

A. What are your general impressions of the app?

B. What do you like or not like about it?

C. What are your thoughts on the design and format?

D. What are your thoughts on the content? Is there anything that needs to come off? Is there anything you feel should be on and is not?

E. Was it easy to navigate and use?

F. Can you think of any issues or difficulties with using the app? If yes then how would you make it easier to use/implement?

G. Do you think the app can help you better manage your illness?

Supplementary Table S4 Interview guide for phase III (interviews with young people)

A. Would you be able to use the app by yourself (without the help of a parent)?

B. What did you think about the reward system?

C. Was it easy to input information for the different sections?

D. Was it quick to find section and information that you were looking for?

E. Did every page and video load quickly?

F. Did each section take an appropriate time to complete?

G. Are the words and instructions clear and easy to understand

    i. Was it clear what you could use each section for?

    ii. Did the buttons in the app work as you expect them to?

    iii. Was it clear how you can save your data?

    iv. Was it clear how you can view your data?

H. Will you use the app regularly in the future? Why or why not?

I. Will this app help you with your arthritis self-management? Why or why not?
