# Supplementary material for: Developing and Evaluating JIApp: Acceptability and Usability of a Smartphone App System to Improve Self-Management in Young People With Juvenile Idiopathic Arthritis
Source: JMIR Mhealth Uhealth. 2017 Aug 15;5(8):e121. doi: 10.2196/mhealth.7229 (PMC5575419; doi:10.2196/mhealth.7229)
Supplement: Multimedia Appendix 2 [file mhealth_v5i8e121_app2.pdf]

	Strongly disagree				Strongly agree
**Usability**					
1) Overall it was easy to use this application.	1	2	3	4	5
2) I was able to complete the tasks quickly using this application.	1	2	3	4	5
3) I felt comfortable using this application	1	2	3	4	5
4) It was easy and quick for me to find the information I needed.	1	2	3	4	5
5) The organization of information on the application screen was clear.	1	2	3	4	5
**Acceptability**					
6) The application looked pleasant (e.g., pictures, words, style etc.)	1	2	3	4	5
7) The application was fun to use.	1	2	3	4	5
8) I found this application useful for me.	1	2	3	4	5
9) Overall, I am satisfied with this application.	1	2	3	4	5
10) I would recommend this application to other young people with arthritis.	1	2	3	4	5

Multimedia Appendix 2: Usability questionnaire
